# Penile Strangulation in a 68 Years Old Male by Plastic Bottleneck: A Case Report

**DOI:** 10.1002/ccr3.73511

**Published:** 2026-09-10

**Authors:** Nafisa Zaman Akhtar, Zakia Akther Zahan, Anika Chowdhury, Zareen Tabassum, Anika Tasnim Khan, Humayra Tabassum, Md. Deluwar Hussen

**Affiliations:** ^1^ Dinajpur Medical College Dinajpur Bangladesh; ^2^ Dhaka Central International Medical College and Hospital Dhaka Bangladesh; ^3^ Shaheed Suhrawardy Medical College and Hospital Dhaka Bangladesh; ^4^ Chattogram Medical College Chattogram Bangladesh; ^5^ Mugda Medical College Dhaka Bangladesh

**Keywords:** case report, constricting device, paraphilia, penile strangulation, plastic bottle neck, urology

## Abstract

Penile strangulation is an uncommon urological emergency. The patient is a 68‐year‐old man with penile constriction caused by using plastic bottles resulting in distal edema. Liston bone cutter was used to remove the object under local anesthesia without open surgery. The urinary and erectile functions were maintained.

## Key Clinical Message

Penile strangulation mostly occurs in younger males with pervertism. Elderly cases like ours are exceedingly rare, especially in conservative developing countries. Superstition drives patients towards traditional healers causing critical delays. Despite late presentations, this case was successfully managed without surgical intervention, preserving both urinary and erectile function.

## 1 Introduction

Self‐inflicted penile strangulation by a number of metallic and non metallic objects (e.g., metallic rings, bottles, hair, thread, rubber bands) is an uncommon urological emergency. Although it can be observed in any age group, occurrences in the middle aged and elderly population are commonly reported [1]. In majority of the cases, patients are found to experiment with these objects to achieve sexual pleasure, prolong penile erection, due to psychiatric illnesses or as a form of paraphilic behavior [2]. The usual presentation is edema but cases may lead to necrosis, sepsis or even gangrene. Although these incidents require prompt removal of the encircling object, there are no specific guidelines to manage these cases. Because management strategies vary from case to case as the constricting objects differ and removal demands use of unconventional equipment and technicality [3].

We herein report a case of a 68 years. old male patient with self‐inflicted penile strangulation by a plastic bottle, resolved by removal of the object and conservative treatment. This case aims to create awareness needed to address the psychological aspect as a causative factor, alleviate the social stigmata around sexual education and management of this rare case.

## 2 Clinical Case Presentation

A 68 years old male was admitted to Male Surgery Ward, Dinajpur Medical College Hospital, Dinajpur, through Emergency Department, with the complaints of pain and swelling of the penis following insertion of a plastic bottleneck. According to the statement of the patient and his attendant, he had a history of repeated engagement in similar activities over the past 5–7 years using multiple objects, including glass bottles and had always been able to remove them by himself without the requirement of hospital admission. On further query, the patient stated that dissatisfaction related to his partner's sexual inactivity led him to seek alternative means of sexual gratification and gradual development of an interest in using inanimate objects to meet his sexual urges in later life rather than during adolescence or early adulthood. There was no history of psychosis, substance abuse, or prior psychiatric illness.

**FIGURE 1:**
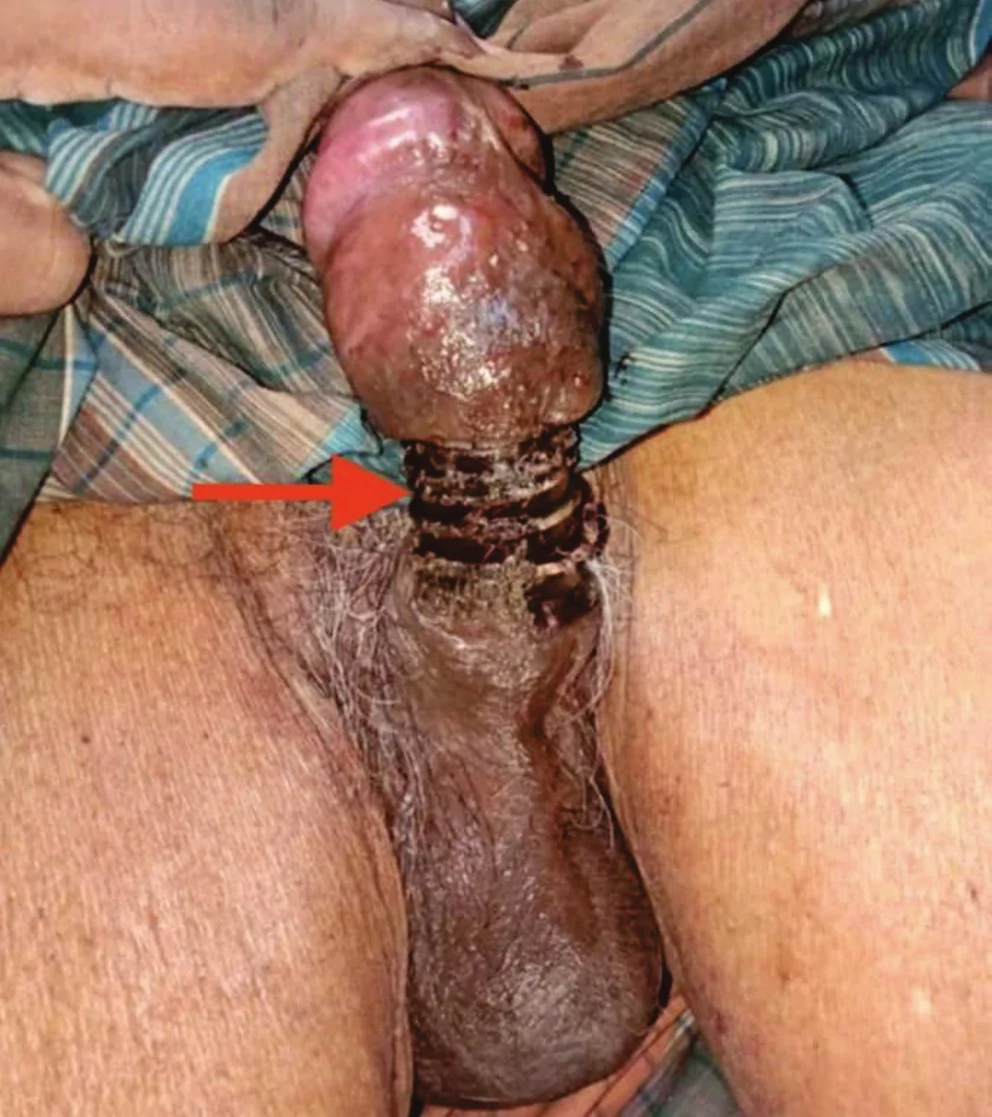
A plastic bottle neck (red arrow) constricting the proximal portion of shaft of penis with marked edema of distal portion.

**FIGURE 2:**
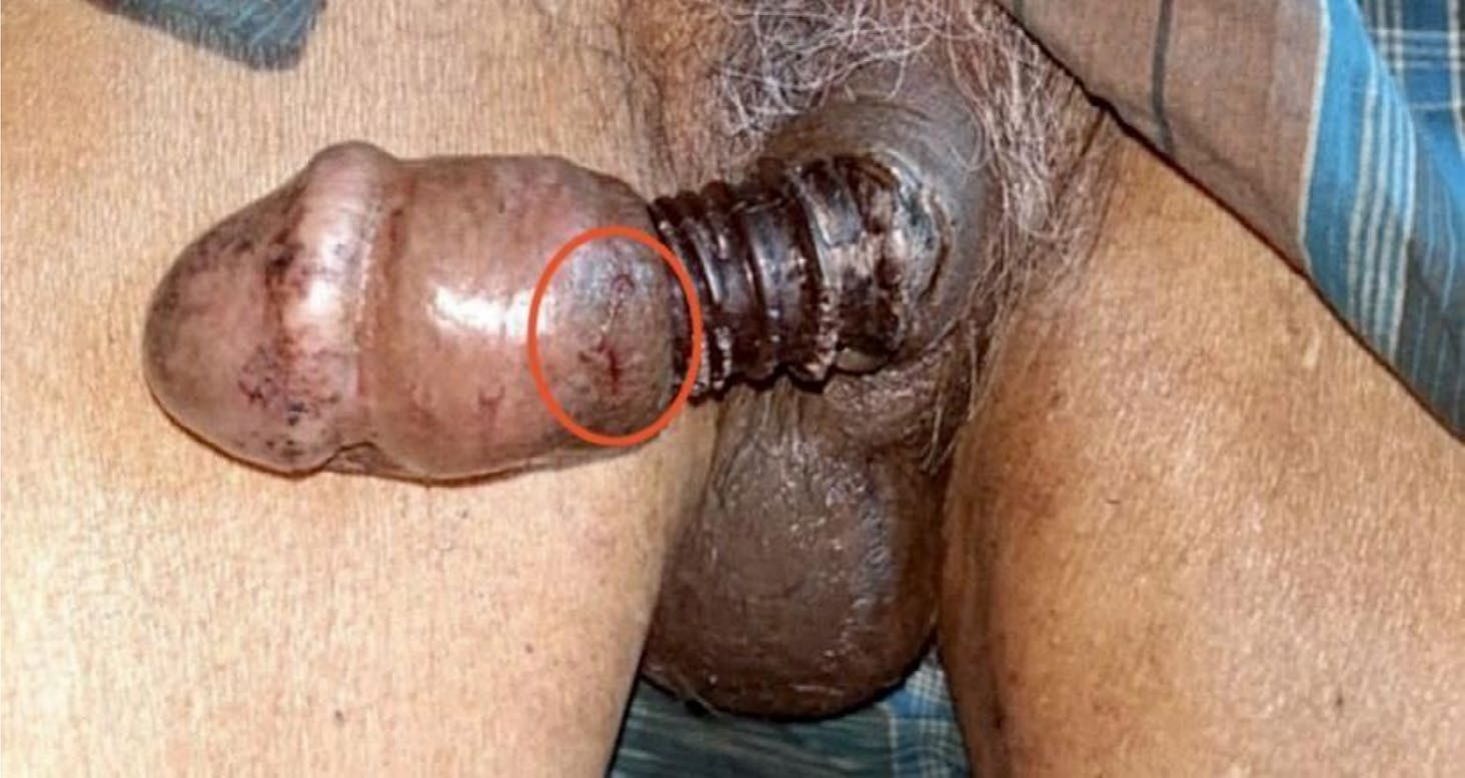
Small superficial skin laceration (red circle) present on dorsal surface of shaft of penis.

On admission, the patient was conscious and well oriented, with a Glasgow Coma Scale score of 15/15. There were no clinical signs of psychosis or intoxication. On general physical examination, he was non‐anemic, non‐icteric. There was no sign of clubbing, cyanosis, koilonychia, or leukonychia. His pulse was 88 bpm, BP was 120/70, respiratory rate was 18 breaths/min, temperature was 98°F. On local examination, a 3–4 cm long plastic bottleneck was found tightly lodged at the proximal portion of the shaft of the penis, with marked distal penile edema. 2–3 small superficial skin lacerations were seen on the dorsal surface of the penile shaft (Figures 1 and 2).

## 3 Investigations

After initial assessment, routine investigations were performed revealing the following findings: *Complete blood count (CBC)*: Hemoglobin (Hb) 13.5 g/dL; White blood cell (WBC) count 5.4 × 10^3^/μL, *Erythrocyte sedimentation rate (ESR)*: 40 mm/h, *C‐reactive protein (CRP), quantitative*: 4.7 mg/dL, *Serum creatinine*: 0.72 mg/dL, *Urine routine and microscopy (R/M/E): Pus cells* 0–1/HPF, *Epithelial cells* 1–2/HPF.

## 4 Treatment

**FIGURE 3:**
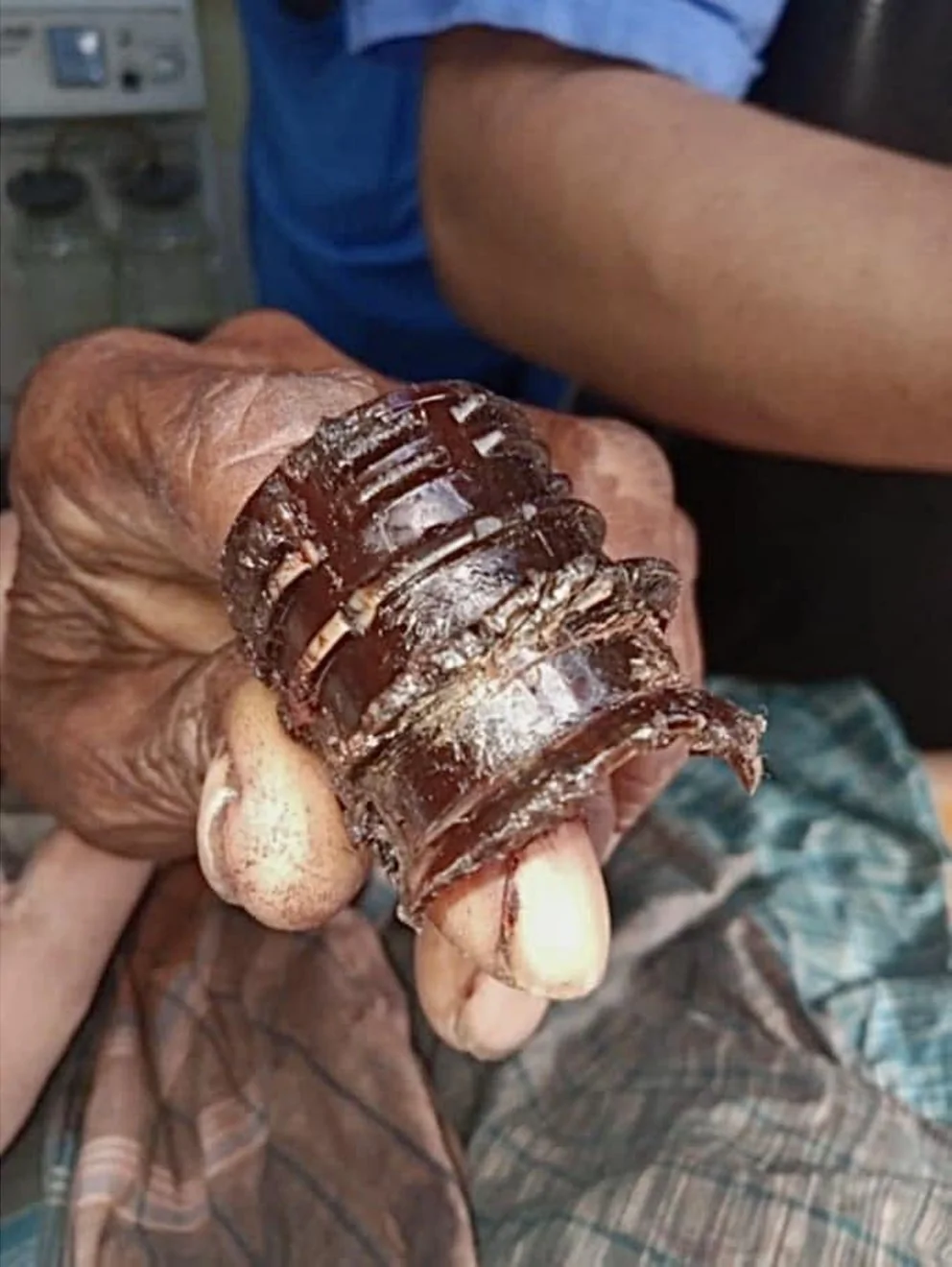
Part of constricting object (plastic bottleneck) after removal.

After initial lubrication‐assisted removal had failed, the patient was taken to the Operative Room (OR) in parallel with routine investigations due to the risk of worsening edema and ischemia, in anticipation of possible surgical intervention. Local anesthesia was administered at the spinal root level. A rough surgical scissor was used to smooth out the sharp edges of the bottleneck. However, to minimize the risk of iatrogenic injury to the penile tissues, a Liston bone cutter was subsequently employed to cut through the bottleneck and relieve the constriction. The constricting object (plastic bottleneck) was successfully removed without the need for open surgery (Figure 3).

## 5 Outcome

Post‐removal assessment revealed no evidence of tissue necrosis or vascular compromise. Both testes were viable and testicular arteries were intact. The motor and sensory functions of the genitalia were preserved. Urology consultation was sought. Urethral catheterization was deliberately avoided to prevent the potential urethral injury in the setting of recent strangulation. Urethroscopy was not available at the time; therefore, the urology team advised suprapubic catheterization only if urinary retention developed, followed by delayed urethroscopy if indicated. The patient was advised to maintain adequate oral hydration and was able to void bladder spontaneously after 7–8 h. Based on clinical findings, the injury was classified as Grade I penile strangulation according to the Bhat et al. classification, characterized by distal edema; and according to the Silberstein classification, this was a low grade injury which is typically treated conservatively without the need for surgical intervention in most cases.

## 6 Follow Up

The patient was discharged after 3–4 days with oral antibiotics and analgesics. On follow‐up after 5 days, the penis appeared normal, and the patient reported proper morning erection. Psychiatric evaluation and psychological therapy were advised for further assessment and counseling.

## 7 Discussion

Penile strangulation caused by metallic rings is a rare acute urological emergency that is associated with attempts to enhance sexual performance, underlying psychiatric disorder, or paraphilic behavior [4]. It requires prompt intervention [4]. Prolonged penile constriction obstructs arterial, venous, and lymphatic drainage and thus compromises arterial blood to the corpora cavernosum, causing compartment syndrome, gangrene, necrosis, and penile amputation [5].

While metallic rings (36%) are most common among adults (mean age 44.2 years). Strangulation in patients over 65 using plastic bottle necks is rare [5]. Our patient's injury was Grade I according to classification. The injury was managed conservatively using a Liston bone cutter avoiding both open surgery. The preservation of erectile, sensory, and urinary functions in this case demonstrates that despite resource constraints, timely management can be highly effective. He attributed his behavior to dissatisfaction with his partner, possibly rationalizing an underlying object fetish. Paraphilic behaviors are often under‐recognized and may remain undiagnosed [6]. Although paraphilic behaviors typically begin in early life, this patient reported onset only 7–8 years ago, highlighting the unusual late presentation. Ultimately, the Bhat grading system can help guide treatment, and early multidisciplinary involvement improves outcome.

## 8 Conclusion

Adult penile strangulation due to the application of a constricting device is a rare urological emergency that needs prompt treatment to avoid irreparable damage in the long term. The timely intervention of skilled doctors with proper methods may restore almost normal physiological functioning and enhance prognosis. In patients with paraphilia and chronic penile strangulation, it is necessary to perform psychological evaluation. This approach increases the maximum chance of successful outcome ensured by multidisciplinary participation.

## Author Contributions

**Nafisa Zaman Akhtar:** conceptualization, investigation, writing – original draft, methodology, writing – review and editing, formal analysis. **Zakia Akther Zahan:** conceptualization, investigation, writing – original draft, writing – review and editing, visualization, methodology, formal analysis. **Anika Chowdhury:** writing – original draft, writing – review and editing, project administration, supervision, data curation. **Zareen Tabassum:** conceptualization, investigation, writing – original draft, writing – review and editing, supervision, formal analysis. **Anika Tasnim Khan:** conceptualization, investigation, methodology, validation, software, formal analysis, data curation, supervision. **Humayra Tabassum:** software, formal analysis, data curation, supervision, validation. **Md. Deluwar Hussen:** conceptualization, funding acquisition, validation, data curation.

## Funding

The authors have nothing to report.

## Supporting information


**Data S1:** ccr373511‐sup‐0001‐Supinfo1.docx.

**Data S2:** ccr373511‐sup‐0002‐Supinfo2.docx.

## Data Availability

The data that support the findings of this study are available from the corresponding author upon reasonable request.
