# Supplementary material for: Penile Strangulation in a 68 Years Old Male by Plastic Bottleneck: A Case Report
Source: Clin Case Rep. 2026 Sep 10;14(9):e73511. doi: 10.1002/ccr3.73511 (PMC13562605; doi:10.1002/ccr3.73511)
Supplement: Supplementary file 2 [file CCR3-14-e73511-s002.docx]

**Author’s information:**

1) Nafisa Zaman Akhtar, MBBS, Dinajpur Medical College and Hospital, Dinajpur, Address: 319, Senpara Parbata, Mirpur-10, Dhaka-1216, Bangladesh. [nafisaz.akhtar49@gmail.com](mailto:nafisaz.akhtar49@gmail.com) , [ORCID ID: 0009-0006-0238-4230](https://orcid.org/orcid-search/search) , +880 1307988393

2) Zakia Akther Zahan, MBBS, Dhaka Central International Medical College and Hospital, Address: Dhanmondi 3/A, Dhaka -1209,Bangladesh. [zakia08jahan@gmail.com](mailto:zakia08jahan@gmail.com) , [ORCID ID: 0009-0009-8631-4257](https://orcid.org/orcid-search/search), +880 1722740244

3) Dr.Anika Chowdhury, MBBS, Shaheed Suhrawardy Medical College and Hospital, Address: Sher E Bangla Nagar , Dhaka – 1207, Bangladesh, [anikachowdhury5894@gmail.com](mailto:anikachowdhury5894@gmail.com) , [ORCID ID: 0009-0006-6220-991X](https://orcid.org/orcid-search/search) , +880 15169833643

4) Zareen Tabassum, MBBS, Dinajpur Medical College and Hospital, Dinajpur, Address: 4/1/B Golden Street, Ring road, Shyamoli, Dhaka-1207, Bangladesh. [ztaunima72@gmail.com](mailto:ztaunima72@gmail.com), +880 1953899591

5) Anika Tasnim Khan, MBBS, Chattogram Medical College and Hospital, Chattogram, Bangladesh, Address: 48 North Mugda, Dhaka-1214, Bangladesh. [anikatasnimkhan195@gmail.com](mailto:anikatasnimkhan195@gmail.com), [ORCID ID: 0009-0005-5431-4229](https://orcid.org/orcid-search/search), +880 1911620209

6) Humayra Tabassum, MBBS, Mugda Medical College and Hospital, Dhaka, Bangladesh, Address: House 378, Road 9/A, Block D, Bashundhara R/A,Dhaka-1229,Bangladesh. [humi.tabassum@gmail.com](mailto:humi.tabassum@gmail.com), [ORCID ID: 0009-0003-0929-421X](https://orcid.org/orcid-search/search) , +880 1325338778

7) Dr. Md. Deluwar Hussen, MBBS, Dinajpur Medical College and Hospital, Dinajpur, Full address: Ruksana Monji, Shantibagh R/A, Sreemangal-3210, Moulvibazar, Sylhet, Bangladesh. [deluwar664@gmail.com](mailto:deluwar664@gmail.com) [ORCID ID: 0009-0002-1518-5805](https://orcid.org/0009-0002-1518-5805) ,+880 1721248551

**Corresponding author:**

Dr.Anika Chowdhury, MBBS, Shaheed Suhrawardy Medical College and Hospital, Address: Sher E Bangla Nagar , Dhaka – 1207, Bangladesh, [anikachowdhury5894@gmail.com](mailto:anikachowdhury5894@gmail.com) ORCID- 0009-0006-6220-991X, +880 15169833643

**Declaration of interest:** All authors confirm they adopt no conflict of interest and would like to support the corresponding author for submission.

**Informed Consent:**

Informed consent was collected from the patient before collecting data and participation was completely voluntary.

**Funding information:**

This research received no funding from any agent in the public, commercial or the hospital authority.

**Data sharing statement:** No separate datasets were generated or analyzed for this case report beyond the clinical details presented in the manuscript. Any additional information, if requested for legitimate academic or clinical purposes, may be made available by the corresponding author upon reasonable request, subject to institutional approval and strict adherence to patient confidentiality and data protection standards. Identifiable information will not be shared, and any disclosure will be limited to fully anonymized content in accordance with ethical guidelines and informed consent provisions.

**Ethical Considerations:** Institutional Review Board approval was not required for this case report in accordance with institutional policies. Written informed consent was obtained from the patient for publication of clinical details and images. The authors declare no conflicts of interest, and all ethical standards were adhered to throughout the preparation of this manuscript.

**Institutional Affiliation:**

Dream USMLE was the primary institution for this research, ensuring ethical guidance, protection of data and compliance with regulations.

**Acknowledgments:**

We are especially grateful to Dr. Abdullah Al Masud, MD, FACP and his organization Dream USMLE, 16523 Wildhorse Creek 63017, Chesterfield, MO, USA, who gave us the technical guidance.

**Registration:**

It’s not applicable for this study.

**Reporting Guideline:**

This manuscript was prepared in accordance with the CARE (CAse REport) guidelines to ensure transparent, complete, and standardized reporting of this case.

Generative AI Use: For paraphrasing of citations and basic structure of outline, actually very limited use of Quillbot has been made and all have been edited properly after review.

**Generative AI Use:** For paraphrasing of citations and basic structure of outline, actually very limited use of Quillbot has been made and all have been edited properly after review.

**Author contributions (CRediT taxonomy):**

1) Nafisa Zaman Akhtar: Conceptualization; Methodology; Investigation; Data curation; Formal analysis; Writing – original draft; Writing – review & editing.

2) Zakia Akther Zahan: Investigation; Data curation; Writing – original draft; Writing – review & editing.

3) Anika Chowdhury: Investigation; Data curation; Writing – original draft; Writing – review & editing; Project administration.

4) Zareen Tabassum: Investigation; Data curation; Writing – original draft; Writing – review & editing.

5) Anika Tasnim Khan: Investigation; Data curation; Writing – original draft; Writing – review & editing.

6) Humayra Tabassum: Validation; Writing – review & editing.

7) Md. Deluwar Hussen: Formal analysis; Validation; Supervision; Writing – review & editing.

All authors read and approved the final manuscript.
